# Polyurethane-based three-dimensional printing for biological mesh carriers

**DOI:** 10.1038/s41598-024-63000-3

**Published:** 2024-05-29

**Authors:** Feng Wang, Lin Hou, Yan-Hui Shan, Zhen-Su Li, Xiao-Feng Yang

**Affiliations:** 1 Department of General Surgery, The First Hospital of Shanxi Medical University, Taiyuan, 030001 Shanxi Province China; 2 The First Clinical College of Shanxi Medical University, Taiyuan, 030001 Shanxi Province China; 3 Department of Urology, The First Hospital of Shanxi Medical University, Taiyuan, 030001 Shanxi Province China

**Keywords:** Medical research, Translational research

## Abstract

Repair and reconstruction of the myopectineal orifice area using meshes is the mainstay of surgical treatment of inguinal hernias. However, the limitations of existing meshes are becoming increasingly evident in clinical applications; thus, the idea of using three-dimensionally (3D)-printed biological meshes was put forward. According to the current level of the 3D printing technology and the inherent characteristics of biological materials, the direct use of the 3D printing technology for making biological materials into finished products suitable for clinical applications is not yet supported, but synthetic materials can be first printed into 3D form carriers, compounded with biological materials, and finally made into finished products. The purpose of this study was to develop a technical protocol for making 3D-printed biomesh carriers using polyurethane as a raw material. In our study: raw material, polyurethane; weight, 20–30 g/m^2^; weaving method, hexagonal mesh; elastic tension aspect ratio, 2:1; diameters of pores, 0.1–1 mm; surface area, 8 × 12 cm^2^; the optimal printing layer height, temperature and velocity were 0.1 mm, 210–220 °C and 60 mm/s. Its clinical significance lies in: (1) applied to preoperative planning and design a detailed surgical plan; (2) applied to special types of surgery including patients in puberty, recurrent and compound inguinal hernias; (3) significantly improve the efficiency of doctor-patient communication; (4) it can shorten the operation and recovery period by about 1/3 and can save about 1/4 of the cost for patients; (5) the learning curve is significantly shortened, which is conducive to the cultivation of reserve talents.

## Introduction

Inguinal hernia is a common disease that can be treated by abdominal surgery, with > 20 million inguinal hernia surgeries per year performed worldwide^1^and > 1.5 million per year in China alone. Inguinal hernia is more common in men and the elderly, the incidence and prevalence of inguinal hernia are not precisely known. The chance of a person having to undergo an inguinal hernia operation during his/her life is quite high, 27% in the case of men and 3% in the case of women. As almost all diagnosed inguinal hernias are operated on, the natural course of an untreated inguinal hernia is scarcely known. Spontaneous recovery has never been described in adults^2^. When intra-abdominal organs or tissues enter the hernia sac, the presence of the hernia ring (neck) may compress the hernia contents and form an incarcerated hernia. If the intestines are involved, it can cause mechanical obstruction of the intestines and produce a series of clinical manifestations and pathophysiological changes. With prolonged compression time, the intestine may develop edema, exudation, and the incarcerated intestines may experience vascular disorders. If not treated in time, this could lead to necrosis and perforation of the hernia contents, leading to severe peritonitis and even life-threatening. Patients with hernia contents entering the scrotum, especially those with hernia contents entering both sides of the scrotum, often have a long medical history and large hernia sac volumes. The increase in the volume of the hernia sac can significantly affect the daily life of these patients^3^. “Tension-free mesh repair” is the industry standard for treating inguinal hernias^4^. Currently, based on the fee standards of tertiary hospitals at the provincial level, the average treatment cost for inguinal hernia is approximately as follows: (1) open surgery: unilateral 6000 RMB, bilateral 8000 RMB; (2) laparoscopic surgery: unilateral 10,000 RMB, bilateral 13,000 RMB. Depending on the reimbursement rates of medical insurance for each patient, the average treatment cost borne by the individual patient is approximately: (1) open surgery: unilateral 2000 RMB, bilateral 2700 RMB; (2) laparoscopic surgery: unilateral 3300 RMB, bilateral 4300 RMB. Calculated based on the average monthly salary of 5670 RMB in the capital city of our province in 2023, the expenditure proportions are as follows: open surgery: unilateral 35.3%, bilateral 47.6%; laparoscopic surgery: unilateral 58.2%, bilateral 75.8%. Undoubtedly, this increases the burden of patients’ lives. And the incidence of complications associated with “the use of mesh” in clinical practice is at 30%, and these complications are mostly related to factors such as mesh material, displacement, curling, contracture and rejection.

The implant materials used for making meshes can be divided into two categories: synthetic materials and biomaterials, which are used for repairing and reconstructing the myopectineal orifice area through different mechanisms. After years of clinical practice, a common belief has developed in the industry that meshes made of biomaterials have significant advantages over meshes made of synthetic materials. Synthetic meshes include non-absorbable, absorbable and composite meshes, and common complications include recurrence and infection. The recurrence rate ranges from 10 to 24%^5^ while the infection rate is in the 0.5–9.0% range^6^, which has led to a bottleneck in the development of hernia surgery methods^7–10^. Biomaterial meshes include xenogeneic acellular dermal matrix and allogenic acellular dermal matrix meshes. Their advantages are: (1) stable physical and chemical properties, (2) non-carcinogenicity, teratogenicity and allergenicity, (3) tension close to that of the tendinous tissue of the abdominal wall muscles, (4) cuttability, degradability and sterilisability and (5) ease of sourcing and usage^11^. Their disadvantages are their soft texture and poor plasticity. When they are used alone, it is not possible to ensure their intimate adhesion to the tissue that is sought to be repaired without changing their own morphology, and they tend to shift as the patient changes position and moves; consequently, the mesh deviates from the site that it seeks to repair, resulting in recurrence and limiting the mesh’s clinical application^12,13^. Intraoperative fixation using staple guns, bioprotein glue, or manual sutures increases the cost of treatment, difficulty of surgery and probability of complications such as intraoperative bleeding, postoperative seroma and intractable pain. Therefore, it is highly desirable to develop integrated three-dimensionally (3D)-printed biomeshes using biomaterials as the implant components and using composite 3D-printed carriers for support; this would allow the mesh to maintain its original form and ensure its perfect fit to the myopectineal orifice area. Not only can such design significantly improve the performance of individual component materials, it also allows to utilise the characteristics of other materials within the mesh to make up for shortcomings, ultimately allowing to keep the mesh in a more suitable state for the body.

**Figure 1:**
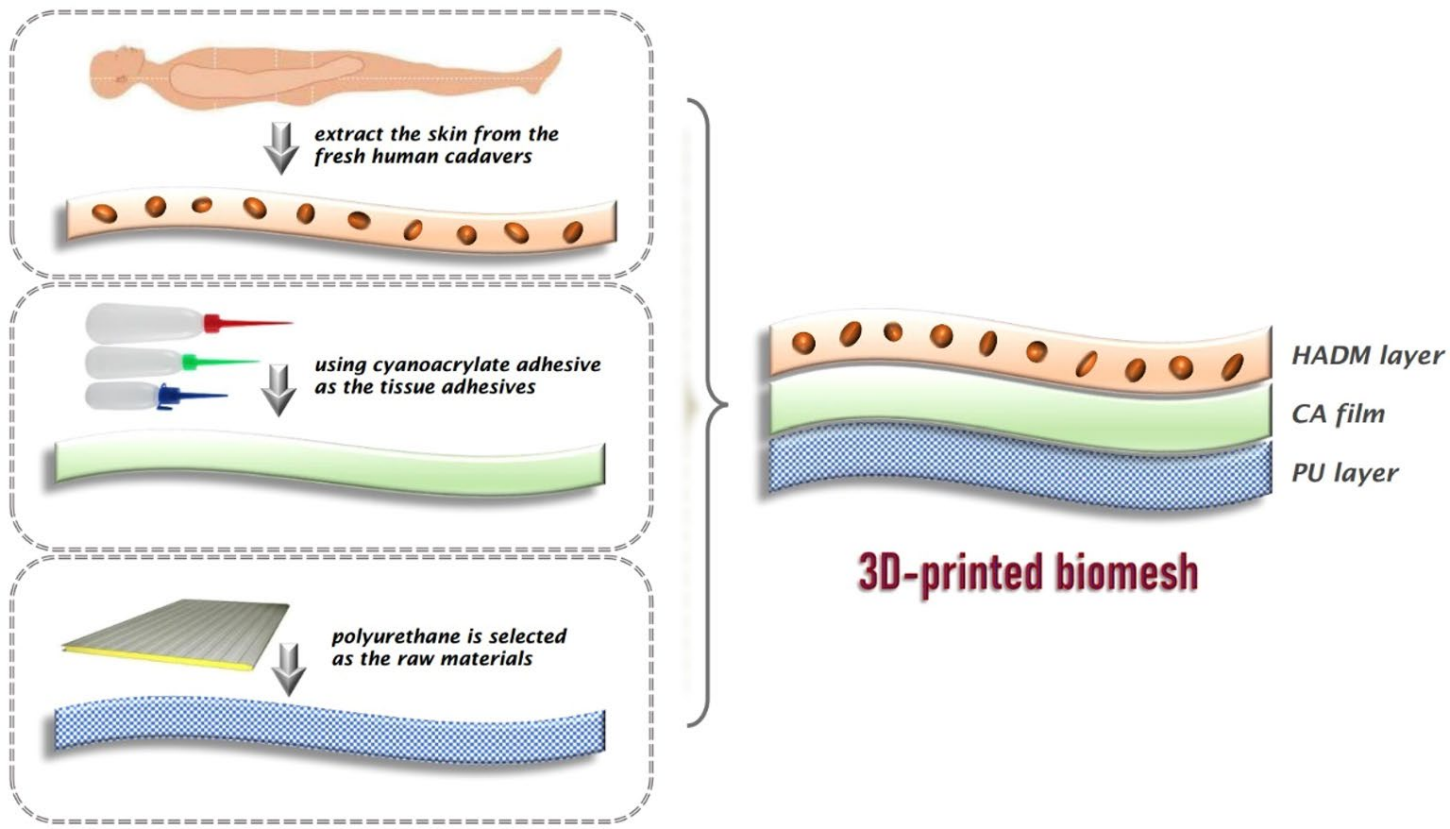
Effect illustration of a 3D-printed biomesh.

Numerous scholars worldwide have been studying 3D-printed meshes. Weisman et al.^14^ investigated the antibacterial and chemotherapeutic properties of these meshes. A research group in Spain studied the meshes’ anti-infection properties and developed meshes with anti-infection ability^15^. However, the above studies have been limited to synthetic materials (without using biomaterials), but at the same time have enabled a systematic theory for clinical applications. Because the existing 3D printing methods cannot be directly applied to biomaterials, for pursuing 3D and biological effects we used the accuracy data information provided by the multi-layer spiral computed tomography (CT) 3D reconstruction technology^16^. The carriers were created using the 3D printing technology, allowing to precisely reconstruct the 3D structure of the myopectineal orifice area. Then, compounding with biomaterials as one, we created 3D-printed biomeshes suitable for clinical applications (Fig. 1). This approach ensured the 3D morphology of the meshes and significantly improved the performance of biomaterials.

3D printing can provide individualized and customized materials and drugs for specific patients to meet the needs of patients in clinical applications^17^. In the future, 3D printing can become a new option for large-scale manufacturing. 3D printing has unique performance characteristics and designs that can improve efficacy and reduce adverse events^18^. The use of patient-specific implants in surgery can also save surgical time and cost^19^. The development of 3D bioprinted organoids marks a crucial advancement, bridging the gap between 2D model studies and animal models, with the potential to eventually replace the latter^20^. However, there are some limitations of 3D printing technology. The biggest challenge is to ensure that the human cells and biomaterials are compatible and can produce stable structures and function properly^20^. Secondly, the application of 3D printing in medicine has not yet been formally formed, and quality control problems will inevitably arise when printing personalized products, and in order to avoid abuse and misuse of products, a guiding framework needs to be developed^18^.And purchasing Purchasing 3D bioprinting equipment in the future is also an ecpensive initial investment^20^.

The 3D printing technology seeks to construct objects based on digital model files by sequentially printing them layer-by-layer using special adhesives. With the introduction of the 3D printing technology into clinical practice, the superiority of its “tailor-made” precision has become increasingly evident. With respect to meshes, 3D printing leads to several advantages: (1) meshes can be designed according to the structure of the individual myopectineal orifice area, thus reducing the risk of recurrence owing to the mesh displacement and curling caused by size mismatches; (2) the approach allows precision integrated moulding of porous hollow structures, according to the specific surgery needs, thus facilitating the actual production needs; (3) the mesh microstructural properties, such as roughness and microporousity, can be regulated to facilitate integration with the body after implantation; (4) the grid size of the mesh can be adjusted to achieve different locations corresponding to different stress-bearing capacities (e.g. the stress-bearing capacity of the central location is approximately threefold that of the other parts; thus, the grid size can be directionally designed to be smaller, improving the stress-bearing capacity).

The objective of this study was to use the 3D printing technology to create biomesh carriers. Ultra-light weight, high mesh porosity, optimal aspect ratio for elastic tension, optimal diameters of pores and small surface area were proposed as the production criteria.

## Methods

### Study design

The following production standards were developed: weight, 20–30 g/m^2^; weaving method, hexagonal mesh; elastic tension aspect ratio, 2:1; diameters of pores, 0.1–1 mm; surface area, 8 × 12 cm^2^. The following data-processing methods were used: 3-matic software was used to draw the overall structure of the surface with moderate thickening; SolidWorks software was used to build the fascia form for designing a hole-like hollow structure; Boolean operations were used for processing the hole-like skeleton structure and for adding a uniformly thin boundary layer, thus converting an open-flow body into a non-flow body. The following materials were used: polyurethane was used as the raw material. The following production parameters were used: layer height, 0.1–0.3 mm; temperature, 200–230 °C; velocity, 30–120 mm/s. Print carrier. For the accuracy comparison test, the error standard of ± 1 mm was set and comparative analysis was performed using a “digital carrier”. Cell viability test. Perform sample size calculation.

### Procedures

The specific procedures are:

1. The choice of construction and the formulation of production standards;
2. Data-processing steps can be divided into 6 steps;
3. Choice of optimal raw material for this study;
4. Select the optimal production parameters, the printed samples were tested for physical property;
5. After the above steps, we proceeded to print the carriers;
6. Accuracy comparison tests that request the error range was ± 1 mm;
7. RTCA technology is used for Cell viability test, observe the effect of the material on the viability of human normal cells;
8. Perform sample size calculation.

### Choice of construction

The following production standards were used: weight, 20–30 g/m^2^; weaving method, hexagonal mesh; elastic tension aspect ratio, 2:1; diameters of pores, 0.1–1 mm; surface area, 8 × 12 cm^2^.

### Data-processing steps

**Figure 2:**
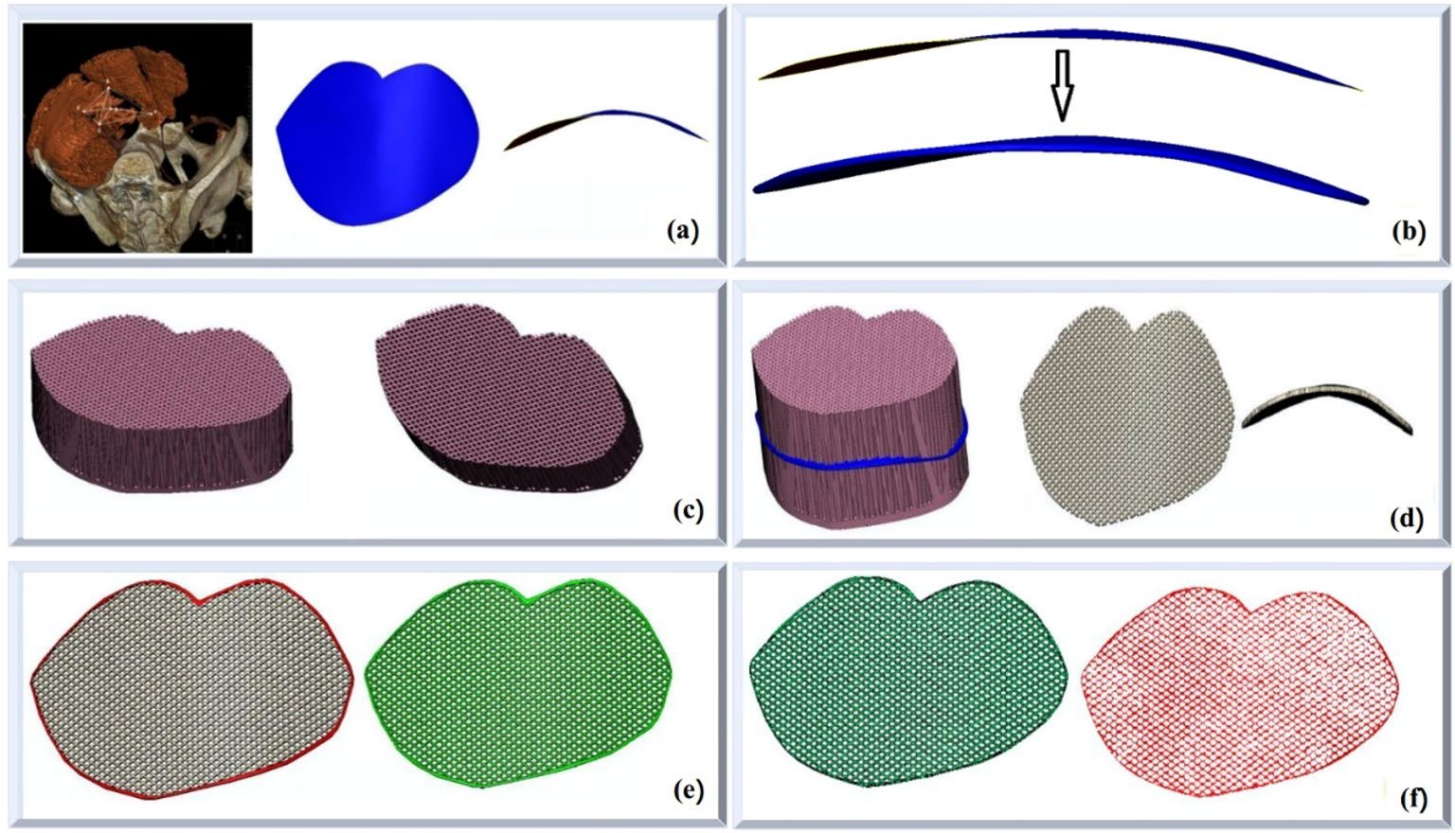
Data processing. (**a**) The overall structure of the curved surface is drawn. (**b**) The thickness is increased. (**c**) The hole-like hollow structure is designed. (**d**) Boolean operations on sets are used for processing the hole-like hollow structure. (**e**) A uniformly thin boundary is added. (**f**) An open-flow body is converted to a non-flow body.

1. Draw the overall structure of the curved surface: 3-matic software was used to draw the overall structure of the curved surface according to the fixed marking points of the myopectineal orifice area. (Fig. 2a)
2. Increase the thickness: because the overall structure of the curved surface was designed as an STL triangular mesh, it was a “surface” rather than a solid body without thickness. The actual structure should be a solid body; thus, it should be moderately thickened, and the thickness was set to 0.5 mm. (Fig. 2b)
3. Design a hole-like hollow structure: based on the designed overall structure of the curved surface, SolidWorks software was used to construct a fascia form and design a hole-like hollow structure by producing a grid-like hollow form. (Fig. 2c)
4. Use Boolean logic to process the hole-like hollow structure: this study used the set difference operations (i.e. A–B and B–A): the designed hollow structure and thickened curved surface sheet structure were subjected to Boolean operations on sets. The pink part was subtracted from the blue part, and then the intersection of the two was taken, yielding the hollow-structure carrier. (Fig. 2d)
5. Add a uniformly thin boundary: a uniformly thin boundary layer was added to the edge of the hollow-structure carrier, for improving the mechanical properties such as strength and elasticity. (Fig. 2e)
6. Convert an open-flow body to a non-flow body: the “digital carrier” has been largely designed, but the current form was an open-flow body, i.e., the curved surface was a regular parameter, and the structure was assembled from a regular curved surface, which needed to be converted to a non-flow body in order to be closer to human anatomy. Thus, Geomagic software was used to process the non-flow body and generate it automatically. (Fig. 2f)

### Choice of material

Polyurethane was selected as the raw material.

### Production parameters

Layer height: regulated in the 0.1–0.3 mm range, with 0.1 mm steps. Temperature: regulated in the 200–230 °C range, with 10 °C steps. Velocity: regulated in the 30–120 mm/s range, with 30 mm/s steps. The other parameters were kept constant while regulating each printing parameter. The printed samples were tested for relative error, surface roughness, material density and tensile strength.

### Carrier printing

After the above steps, we proceeded to print the carriers. In this study, FDM 3D printing technology was adopted and the JG MAKER A-8 3D printer was used.

### Accuracy comparison tests

The printed carriers were placed under CT for thin-layer scanning and the images obtained were imported into MIMICS 19.0 software in the DICOM format for 3D reconstruction. STL files were obtained after pre-processing the images using techniques such as smoothing, noise reduction and determination of the segmentation threshold. The images were then imported into Geomagic software for co-location matching with the previously designed “digital carrier”, i.e. the data obtained after scanning the carriers were located at the same position in the same coordinate system as the original data, and comparative analysis was performed with Geomagic software. Referring to the YY/T 0640-2008 General Requirements for Passive Surgical Implants, the error range was ± 1 mm, complying with clinical-setting requirements.

### Cell viability test

**Figure 3:**
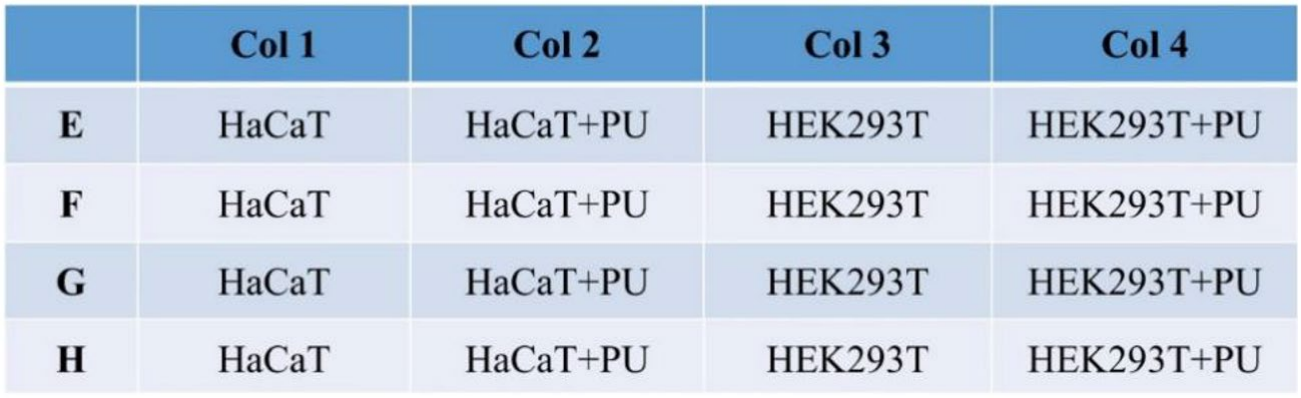
Experimental layout.

RTCA technology is used for detection: add 1 ml trypsin solution containing EDTA (T25 culture flask) into the culture dish, place it in 37 °C incubator for 2–6 min, and then add appropriate amount of culture medium to prepare the cell suspension with cell concentration of 5 × 10^4^ cells/ml. Add 50 µl culture medium into the E-Plate 16 detection plate well and place it on the RTCA instrument. Select the experimental type “propagation/cytotoxicity”, set the resistance value to be detected every 6 h and detect the cell viability. Take out the detection plate and add 100 µl cell suspension into the hole, so that the number of cells in each hole is 5 × 10^3^ cells/well, as shown in the experimental layout. After 30 min at room temperature, the detection plate was placed on RTCA instrument for real-time dynamic monitoring of cell proliferation. After 3 h, add 0.3 × 0.3 cm^2^ sterile polyurethane material into the hole to continue monitoring, and observe the effect of the material on the viability of human normal cells HaCaT and HEK293T cells (Fig. 3).

#### Sample size calculation

The sample size was calculated using PASS 2021, v21.0.3. Information about the population rate was obtained through pre-experiments.

## Calculation

### Calculation of production parameters

Six result data were measured for each process parameter. The experimental results are $x = \overline{x} \pm \overline{u}$ (unit), $x$ is the physical quantity to be measured, $\overline{x}$ is the arithmetic mean of the physical quantity to be measured, and $\overline{u}$ is the combined standard uncertainty. All measurement methods are direct measurement $u = \sqrt {u_{A}^{2} + u_{B}^{2} }$, $u_{A}$ is Type A uncertainty, also known as statistical uncertainty, which refers to the uncertainty that can be calculated by statistical methods. Due to the accidental effect, the data of repeated measurements are scattered and obey the normal distribution law. The confidence of industrial statistics is P = 95%, n = 6, and the degree of freedom (n − 1) df = 5, the table shows t = 2.570.

$$u_{A} = t\sqrt {\frac{{\mathop \sum \nolimits_{i = 1}^{n} \left( {x_{i} - \overline{x}} \right)^{2} }}{{n\left( {n - 1} \right)}}}$$

$u_{B}$ is Type B uncertainty and can be considered as the maximum error during normal use of the instrument.

### Calculation of cell viability test

The statistical description of the data were expressed as means ± standard deviations. The effect of polyurethane material on cell viability were compared using repeated measures ANOVA.

## Results

Following the regulation criteria, the optimal layer height was 0.1 mm; the optimal temperature interval was 210–220 °C and the optimum velocity was 60 mm/s.

### Print carriers

**Figure 4:**
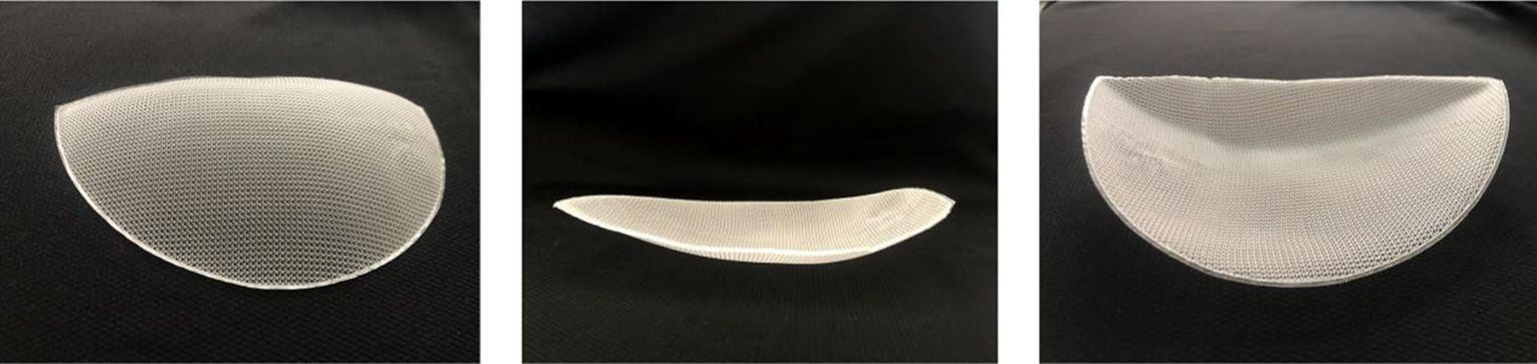
Print carriers.

As shown in the figure below, the figure is the carriers printed out in this study. (Fig. 4).

### Mechanical properties of the printed carrier

#### Structural analysis of the printed carrier (SEM)

**Figure 5:**
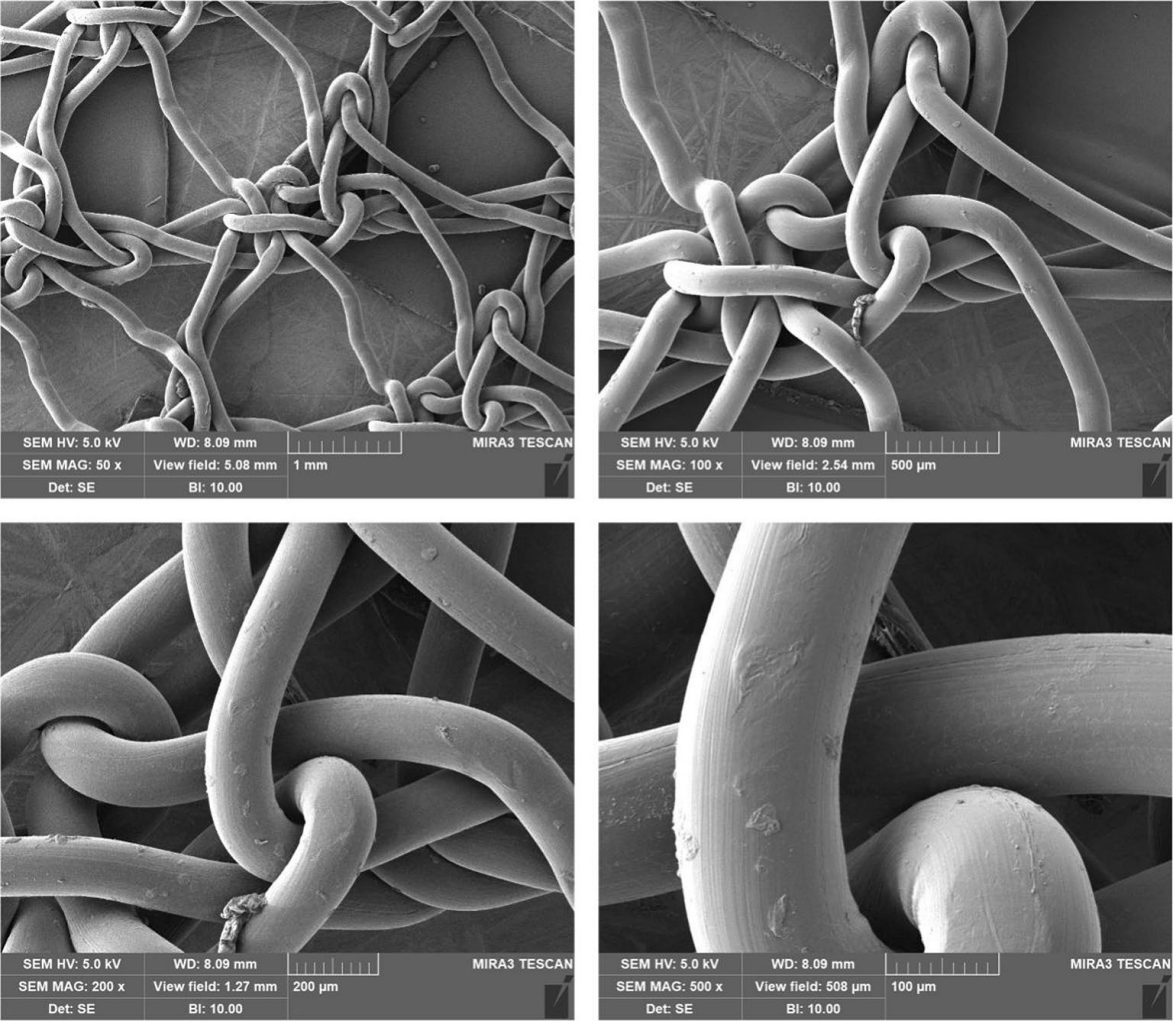
Structure of printed carrier under SEM.

As shown in the figure below, the figure is the structure of the printed carrier. (Fig. 5).

#### Accuracy comparison tests

**Figure 6:**
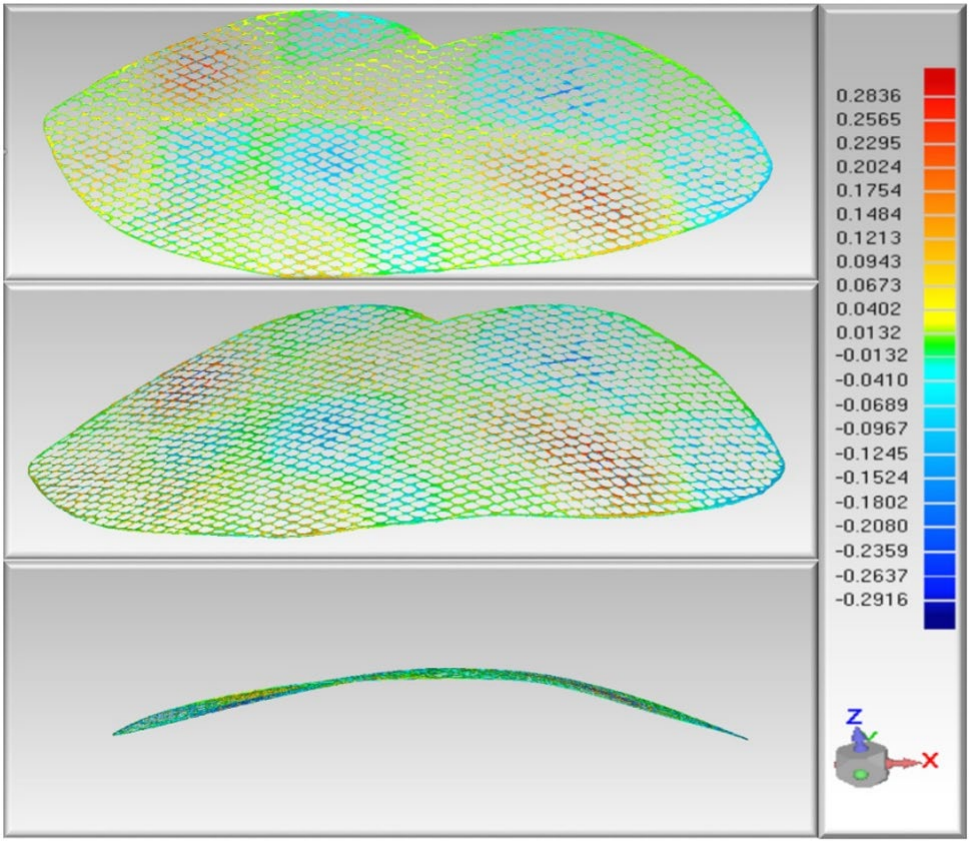
Accuracy comparison tests. *Note* the red and blue parts of the scale area represent the range of the upper and lower deviation areas, respectively: the darker the red, the greater the upper deviation; the darker the blue, the greater the lower deviation; the smaller the deviation in the middle part, the lighter the corresponding colour.

The accuracy of the printed carriers was compared with that of the previously designed “digital carrier”, and the error range was − 0.2788 to 0.2836 mm, satisfying the design and production requirements. (Fig. 6).

#### Cell viability test

**Figure 7:**
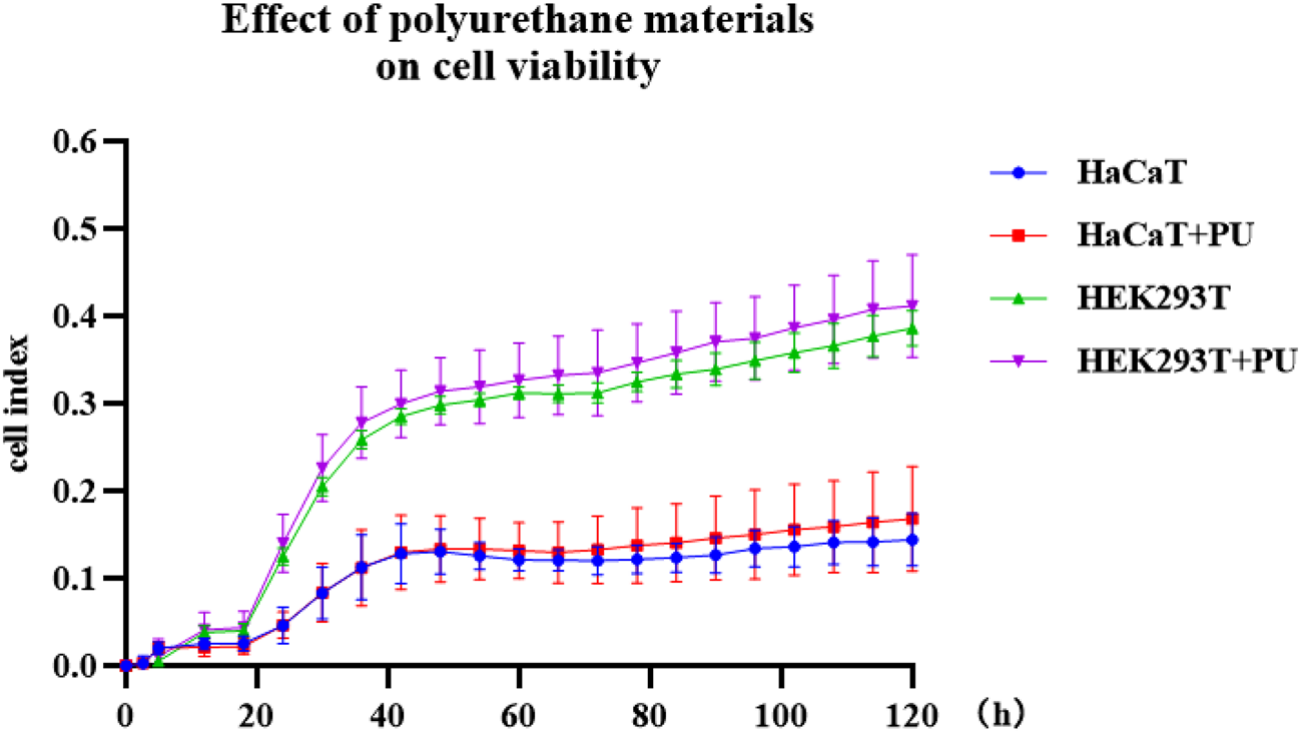
Effect of polyurethane materials on cell viability.

We used RTCA technology for cell viability testing. Figure 7 shows the effect of polyurethane material on resistance values of human normal cells HaCaT and HEK293T cells, which were analysed by two-way repeated measures ANOVA. No difference was found between the HaCaT and HaCaT + PU group (F = 0.642, P > 0.05), but an effect of time was determined (F = 27.739, *P* < 0.05), which was not influenced by interaction of group and time (F = 0.648, *P* > 0.05). And no difference was found between the HEK293T and HEK293T + PU group (F = 0.925, *P* > 0.05), but an effect of time was determined (F = 685.844, *P* < 0.05), which was not influenced by interaction of group and time (F = 0.930, *P* > 0.05). The results showed that there was no statistical difference in the cell resistance values after adding the polyurethane material, which proved that the polyurethane material had no effect on cell viability and was non-toxic to cells and could be used for the production of 3D-printed biomesh carriers.

#### Sample size calculation

**Figure 8:**
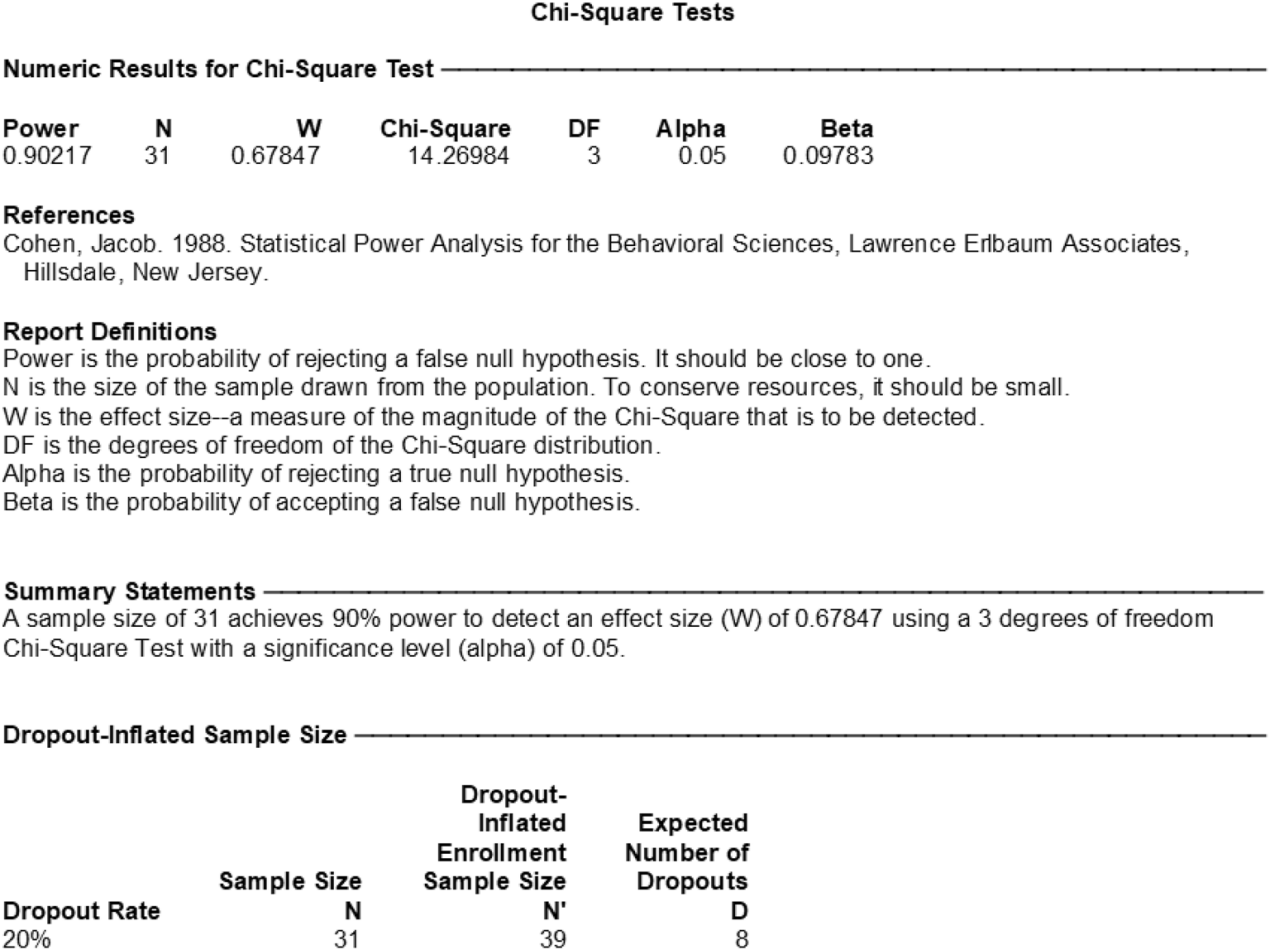
Sample size calculation.

As shown in Fig. 8, the population rates of 4 groups were 25%, 12.5%, 100% and 37.5%, respectively. The sample size ratio of 4 groups was 1:1:1:1. Based on a level of significance of 0.05, a power of 90%, and an effect size of 0.678467, a minimum total sample size of 32 samples (8 in each group) was determined to be sufficient.

## Discussion

The production of 3D-printed biomesh carriers was modified according to the standards of meshes made from synthetic materials. The main assessment criterion was the suitability for the bodily structure, namely improving the compliance between the web-like scar tissue formed by the mesh and the human abdominal wall after fusion of the mesh with the body, to ensure the following production criteria: ultra-light weight, high porosity, optimal aspect ratio for elastic tension, optimal diameters of pores and small surface area.

The meshes made from synthetic materials weigh 35–100 g/m^2^, their thickness is in the 0.1–0.6 mm range, and they can be categorised into light-, medium-, and heavy-weight meshes^15^. This study demonstrated that carriers with weights in the 20–30 g/m^2^ range and thickness of 0.5 mm can maintain 3D structures without deformation when subjected to pressures of ≤ 10 g by biomaterials in the same area.

The weaving pattern and the meshing method should be carefully considered: hexagonal weaving is the most suitable with respect to the structure of the human abdominal wall and exhibits the highest porosity; the method affects the aspect ratio of elastic tension and surface area, which determines not only the overall mechanical properties, but also whether the stress under pressure is isotropic or anisotropic^21^.

Tensile Modulus refers to the elasticity of materials in tension, specifically the elastic modulus under normal stress. In TEP (Total extraperitoneal) surgery, the area where the mesh is placed is the myopectineal orifice area, that is, between the abdominal wall tissue and the peritoneum. The stress it is subjected to mostly comes from the pressure in the abdominal cavity, which is normal stress for the mesh. At this time, the elastic modulus of the mesh is the tensile modulus. In the field of hernia surgery, since the abdominal pressure is variable and the abdominal wall has motor function, it is required that the mesh also has certain elasticity and can comply with the changes in the movement of abdominal wall muscles. The longitudinal mobility of the abdominal wall muscles is higher than that of the transverse, so the longitudinal elasticity of the mesh should be higher than the transverse elasticity. The polyurethane material used in the experiment is a commonly used clinical material, and its tensile strength (28–42 MPa) far exceeds the tensile strength of abdominal wall tissue, so the tensile strength of the material when it reaches the failure stress is not considered, and only the tensile modulus of the material structure is considered. In practical application, the focus of mesh on elasticity is on the aspect ratio of elastic tensile force, which is currently recognized by the industry as 2:1, that is, the aspect ratio of tensile modulus is 2:1. Under the premise of material determination, only the mesh structure can be changed to change the tensile modulus. Therefore, the mesh structure is selected as the mesh size with the longitudinal to transverse force ratio of 2:1, which is used as the elasticity standard in the mesh carrier design.

The diameters of pores of mesh are divided into 5 types: micropore (< 0.1 mm), small pore (0.1–0.6 mm), medium pore (0.6–1 mm), large pore (1–2 mm) and giant pore (> 2 mm)^22^. The microporous mesh has good anti-adhesion effect; The small and middle pore mesh have high strength, can withstand large abdominal pressure impact, and are not easy to deform and shift to ensure the quality of repair; large pore mesh is easy to fuse with the surrounding tissues, but the strength is poor, the abdominal pressure of normal adults can reach 170 mmHg during severe cough or jumping, easily exceeds its limit value resulting in hernia recurrence^23^.In the design of mesh carrier, the parameters of diameters of pores are modified according to the standard of synthetic material mesh commonly used in clinic, such as Medtronic mesh: PP1509G; BARD mesh: 0112650. Since the role of the mesh carrier is to carry rather than repair, the strength factor of the mesh carrier should be given priority, and the parameters of diameters of pores should be selected for small and medium pores. In practical application, the stress at the center of the hernia is about 3 times higher than that at the other parts, and the weaving process can be used to regulate the diameters of pores to achieve different stress bearing abilities corresponding to different positions: the stress bearing ability is enhanced by directionally designing the center position into small pores (0.1–0.6 mm), and the periphery is designed as middle pores (0.6–1 mm) to withstand the general pressure of the abdominal cavity, similar to “spider web”.

The study parameters were: weight, 20–30 g/m^2^; weaving method, hexagonal mesh; elastic tension aspect ratio, 2:1; diameters of pores, 0.1–1 mm; surface area, 8 × 12 cm^2^.

The carrier material should have good plasticity, compatibility and tensile strength. At present, the commonly used medical materials in clinical practice are polycaprolactone (PCL), polylactic acid (PLA) and polyurethane (PU). PCL is characterised by its good degradability and strong affinity. Recently, it has been rapidly adopted in the research field of medical degradable materials and drug sustained-release systems; PLA is a biodegradable material with excellent performance and is widely used in biomedical applications^24^, such as drug release^25^, tissue engineering^26^, implant materials^27^ and artificial skin^28^. Not only does PU offer the above-mentioned advantages, but it is also uniquely flexible, assuming the original form of the finished product, which becomes progressively softer after implantation, allowing for an increasingly comfortable recovery, which is the most desired advantage in the production of implant materials.

Many scholars have made a comparative analysis between polyurethane and conventional mesh materials such as polycaprolactone (PCL), polylactic acid (PLA) and other materials^29^. They believe that polyurethane has higher advantages in the biocompatibility, mechanical properties and clinical performance and other advantages than conventional mesh materials, especially in the field of medical 3D printing research is far-reaching and widely used: medical grade thermoplastic polyurethane has also been successfully printed using bioplotting to create 3D‐printing tracheal scaffolds^30^. Pyo et al. have printed high-resolution 3D biomimetic structures, along with high cell viability on the printed structures^30^.Polyurethane materials have been successfully 3D-printed into specific implants using low temperature deposition printing, has been successfully utilised in the field of vascular tissue engineering^30^.In terms of mechanical properties, it can be seen from Table 1 that the tensile strength, tensile elongation, seam strength, ultimate tensile strength, tear resistance and other data of 3D-printed biomesh carriers are significantly superior to the mechanical properties of conventional mesh materials. Polymers made of polyurethane proved to be highly deformable and to have a low elastic modulus, giving it good mechanical properties for soft tissue engineering applications^31^.

**Table 1:** Mechanical properties of the printed carrier.

Technical indicator	Requirement	Test value
Tensile strength	Tensile strength of rehydrated mesh > 0.16 MPa	0.36 MPa
Tensile elongation	Tensile elongation of rehydrated mesh ≥ 10%	33.56%
Seam strength	≥ 16N	23.9 N
Ultimate tensile strength	No fracture under a pressure of ≥ 20 kPa	36.18 Kpa
Tear resistance	Parallel: ≥ 16 N and perpendicular: ≥ 16 N	42.84 N and 41.78 N

In the course of the study, according to the mechanical characteristics, structural characteristics and production standards of clinical repair materials used at present, we consulted relevant literature of materials such as Poly (caprolactone), Poly (lactic acid), Poly (propylene fumarate)^29^and decided to use polyurethane as raw material. Meanwhile, by reviewing various printing technologies such as Fused Filament Fabrication (FFF), Inkjet Printing, Stereolithography (SLA), Low Temperature Deposition^30^, we ultimately selected Frequency-division multiplexing (FDM).

In future research, we will continue to seek new materials that are more suitable for 3D printing by considering factors such as biocompatibility, mechanical strength, and ease of fabrication. At present, the biomimetic smart materials we have learned about are: lectroactive polymers (EAPs), Shape memory polymers (SMPs), Liquid crystal elastomers (LCEs), Shape memory alloys (SMAs)^32^. In the field of healthcare, biomimetic smart materials are revolutionizing tissue engineering and regenerative medicine^33^.

Because the research of 3D-printed biomesh carriers has not yet entered the clinical implementation stage, its clinical performance is still unknown and cannot be compared with traditional mesh materials, but this project is what we plan to do next.

During the design of the technical protocol, the printing layer’s height, temperature and velocity play decisive roles in determining the printing quality:

Layer height is the key to controlling the printing quality, because the smaller the layer height, probably the better the quality. Analysis of the results in Table 2 shows that the relative error and surface roughness of the printed samples were positively correlated with the layer height; the material density was negatively correlated with the layer height, because the smaller the gap between the printing platform and the print head, the tighter the prints, and vice versa. However, overall, the material density varied little; the tensile strength decreased as the layer height increased, owing to the fact that with more printed layers, more layers adhered to each other, increasing the adhesion. The optimal data at this point is the most suitable parameter, the optimal layer height in the present study was 0.1 mm.

**Table 2:** Effect of printing layer height, temperature and velocity.

Parameters parts quality	Relative error ε	Surface roughness Ra, μm	Density D, g/cm^3^	Tensile strength σ, MPa
*l* = 0.1 mm	0.022 ± 0.009	9.2 ± 0.9	1.21 ± 0.04	28 ± 1.2
*l* = 0.2 mm	0.035 ± 0.008	11.1 ± 0.7	1.18 ± 0.05	25 ± 0.9
*l* = 0.3 mm	0.049 ± 0.010	14.56 ± 1.2	1.08 ± 0.03	22 ± 1.3
*t* = 200 °C	0.05 ± 0.007	10.1 ± 1.3	1.15 ± 0.08	28 ± 0.9
*t* = 210 °C	0.03 ± 0.004	8.4 ± 0.9	1.20 ± 0.06	32 ± 0.7
*t* = 220 °C	0.04 ± 0.005	9.5 ± 0.6	1.16 ± 0.07	26 ± 0.8
*v* = 30 mm/s	0.014 ± 0.005	9.7 ± 0.5	1.21 ± 0.05	31.6 ± 0.7
*v* = 60 mm/s	0.019 ± 0.004	11.2 ± 0.7	1.18 ± 0.06	28.5 ± 1.1
*v* = 90 mm/s	0.034 ± 0.005	12.6 ± 1.1	1.15 ± 0.06	26.8 ± 0.7
*v* = 120 mm/s	0.062 ± 0.011	14.5 ± 1.6	0.95 ± 0.07	25.6 ± 1.4

*l* layer height, *t* temperature, *v* velocity.

Temperature is another important factor. Analysis of the results in Table 2 shows that the smallest relative errors and the lowest surface roughness were obtained for the samples printed at temperatures in the 210–220 °C range; the material density theoretically increased with temperature but exhibited no particular behaviour in the above range of temperatures. The tensile strength changed with temperature because different materials exhibit different rheological mechanisms in the molten state, which can result in differentiation between materials and batches of materials. The temperature of the print head determines the properties of 3D printing, such as sustainability, accumulation and adhesion, which can directly affect the accuracy and mechanical properties of the printed samples. Factors such as insufficient dissolution of the material and excessive material viscosity at too low temperatures can slow down the extrusion velocity of molten material, resulting in the reduced adhesion strength of the adhered layer; at too high temperatures, the fluidity of the molten material increases, slowing the cooling and inter-layer solidification, and resulting in the samples’ misaligned adhesion. The experimental data shows that the temperature is optimal at 210 °C, but in practice, although the printer nozzle is set at 210 °C, the printer cannot ensure a constant temperature environment, and the 210–220 °C interval is the most suitable parameter compared with the front and back interval data. After comprehensive consideration, the optimal temperature range in the present study was 210–220 °C.

Finally, velocity is yet another important factor. Analysis of the results in Table 2 shows that as the velocity increased, the relative error of the printed samples progressively increased, with corresponding deterioration in the surface roughness and tensile strength. However, printing velocity essentially did not affect the density of the material and did not change much during the printing process. In the present study, for too high printing velocities, the reduced heating time of the molten material in the flow channel decreased its own plasticisation. Furthermore, since uneven crystallisation already existed when the material was extruded from the print head, it led to uneven shrinkage and deformation of the crystallised material. When the printing velocity was too slow, the cooling time of the molten material increased, which negatively affected inter-layer bonding and fusion. Longer crystallisation times and excessive crystallisation increased the material’s shrinkage. Uneven cooling and shrinkage between the layers slowed the crystallisation of lower levels and promoted the crystallisation of upper layers, eventually resulting in severe warping and deformation of the crystallised material. The experimental data shows that the optimum speed is 30 mm/s, but the fuse is easy to accumulate on the nozzle at this time, resulting in irregular bulge on the product surface occasionally, resulting in a yield of 85.3% and a production time of 2h31 min; while the yield is 97.2% and a production time of 1h48 min at 60 mm/s. After comprehensive consideration, the optimal velocity in the present study was 60 mm/s.

We refer to the verification method of Hernandez-Cordova et al.^31^, the 3D printed samples (4 groups) were scanned by the SHINING RobotScan structured-light 3D scanner, which has the advantages of high precision, high resolution and good stability. The workflow includes setting robot paths, intelligent scanning, automatic detection, and generating customized reports automatically. Import the resulting point cloud data into the Geomagic software for comparing design data with sample data through deviation analysis.

Considering the future clinical application, we attempted to sterilize the PU carrier using Hydrogen Peroxide (H_2_O_2_) low temperature plasma. The PU carrier was placed in a sterilizer with an H_2_O_2_ concentration adjusted to > 55% at a temperature of 45–55 °C for a 50-min short cycle. H_2_O_2_ was vaporized, dispersed, and penetrated the PU carrier in a vacuum chamber to kill some of the surface microorganisms on the carrier. In addition, high-frequency voltage was applied to generate a high-frequency electric field to ionize the H_2_O_2_ gas and activate a plasma system, thereby exerting multiple synergistic bactericidal effects. Next, the plasma system rapidly dissociated the H_2_O_2_ on the surface of the PU carrier into water and oxygen, eliminating any residual substances. The efficacy of sterilization was monitored in accordance with the GB 15981-2021 standard “Evaluation method for the efficacy of sterilization of disinfection equipment.” Chemical and biological detection systems included a chemical indicator strip inside the package and chemical indicator tape outside the package that changed from orange before sterilization to yellow after. The biological indicator was a self-contained culture tube containing 106 CFU/mL of Geobacillus stearothermophilus (ATCC7953) and an ampoule of culture medium, which was removed after completion of the sterilization cycle, incubated at 55 °C, and observed after 24 h. The indicator remained purple if sterile and changed to yellow in the presence of bacterial growth, enabling confirmation of sterilization^34^.

The clinical application of this technology is very wide: (1) applied to preoperative planning: at present, in the treatment of inguinal hernia, the specific data of the structure of the surgical area cannot be obtained by preoperative examination, especially the anatomical structure of the site that has undergone multiple operations is confusing, the fine structure is poorly displayed. Only rely on the experience of the surgeon to prepare the mesh preoperatively, and then repeatedly cut and integrate and measure the matching degree with the myopectineal orifice area during the operation, which is easy to lead to the aggravation of the tissue damage in the surgical area, the increase of bleeding volume, the increase of exogenous infection rate and the extension of the operation time. The 3D-printed carriers have a high degree of matching with the myopectineal orifice area, which can restore its three-dimensional structure, visually display the location and size of the defect and the anatomical relationship between adjacent important blood vessels, nerves and tissues. The surgeon can design a detailed surgical plan based on this, such as the surgical approach, the scope of the surgical field and the emergency plan, and can simulate the surgical process before surgery to reduce the surgical risk. (2) Applied to special types of surgery: the structure of the myopectineal orifice area will change with the age of patients in puberty. At present, the meshes used in the clinic are made according to adult data. Intraoperative cutting and integration will destroy the original structure and lead to high postoperative recurrence rate, and even affect the reproductive function of some male patients, resulting in azoospermia. The 3D-printed carriers, which are accurately made in combination with the characteristics of individual development, can significantly reduce the recurrence rate of such patients, especially the minimal interference to the reproductive system, and significantly improve the quality of life after recovery. The treatment of recurrent and compound inguinal hernias has been a difficult problem, and existing meshes are only suitable for routine cases and require repeated cutting, integrating and matching during surgery, which increases the incidence of various complications. 3D-printed carriers, on the other hand, can fully fit into the patient’s surgical area, reducing the incidence of complications. (3) Applied to doctor-patient communication: in the past, doctor-patient communication could only be based on examination data and experience, and the effect was not satisfactory due to the difference in understanding. Now, 3D-printed models can provide accurate information, a comprehensive understanding of anatomical structures and surgical procedures, and both sides can more clearly clarify the surgical plan^35^ and significantly improve the efficiency of doctor-patient communication. (4) Applied to inpatient management: existing meshes often lead to prolonged hospitalization and increased treatment costs due to the occurrence of various complications and the use of additional consumables. However, 3D-printed carriers have high matching degree and good compatibility with receptors and can shorten the operation and recovery period by about 1/3; the production cost per unit is the same as the batch, which can save about 1/4 of the cost for patients and significantly optimize the management. (5) Applied to clinical teaching: in the past, it was mostly theoretical and experiential teaching, and beginners had no intuitive experience and practical operation opportunities, resulting in relatively difficult mastering of such operations. Applying 3D-printed models to clinical teaching can intuitively understand the structure of the operation area, the operation process and the method of use, the learning curve is significantly shortened^19^, which is conducive to the cultivation of reserve talents.

## Conclusion

In the process of making 3D-printed biomesh carriers, the best technical protocol with the optimal parameters was: raw material, polyurethane; weight, 20–30 g/m^2^; weaving method, hexagonal mesh; elastic tension aspect ratio, 2:1; diameters of pores, 0.1–1 mm; surface area, 8 × 12 cm^2^; 3-matic software, SolidWorks software and Boolean operations were used for formulating the production process; the optimal printing layer height, temperature and velocity were selected as the production parameters. The above optimal choices were combined to formulate the best technical protocol for producing carriers for 3D-printed biomeshes. By regulating the 3D printing layer’s height, temperature and velocity within a certain range, and setting the corresponding optimal parameters to 0.1 mm, 210–220 °C and 60 mm/s, we successfully produced high-precision carriers (error range, − 0.2788 to 0.2836 mm) outperforming the previously designed “digital carrier”. The process and the results obtained using this technical protocol fulfilled the design and production requirements.

## Data Availability

The datasets used and/or analysed during the current study available from the corresponding author on reasonable request.
